# Unpredicted Concentration-Dependent Sensory Properties of Pyrene-Containing NBN-Doped Polycyclic Aromatic Hydrocarbons

**DOI:** 10.3390/molecules27010327

**Published:** 2022-01-05

**Authors:** Hang Xiao, Tao Li, Xiao-Li Sun, Wen-Ming Wan, Hongli Bao, Qingrong Qian, Qinghua Chen

**Affiliations:** 1 Fujian Key Laboratory of Pollution Control & Resource Reuse, Engineering Research Center of Polymer Green Recycling of Ministry of Education, College of Environmental Science and Engineering, Fujian Normal University, Fuzhou 350007, China; QBX20210117@yjs.fjnu.edu.cn (H.X.); qsz20191180@student.fjnu.edu.cn (T.L.); qrqian@fjnu.edu.cn (Q.Q.); cqhuar@126.com (Q.C.); 2 State Key Laboratory of Structural Chemistry, Key Laboratory of Coal to Ethylene Glycol and Its ReLated Technology, Center for Excellence in Molecular Synthesis, Fujian Institute of Research on the Structure of Matter, Chinese Academy of Sciences, 155 West Yangqiao Road, Fuzhou 350002, China; hlbao@fjirsm.ac.cn; 3 State Key Laboratory of Molecular Engineering of Polymers, Fudan University, Shanghai 200438, China

**Keywords:** NBN-doped polycyclic aromatic hydrocarbon, grinding enhanced emission, fluoride ion detection, turn-on type luminescent, artificial light-harvesting film

## Abstract

Pyrene molecules containing NBN-doped polycyclic aromatic hydrocarbons (PAHs) have been synthesized by a simple and efficient intermolecular dehydration reaction between 1-pyrenylboronic acid and aromatic diamine. Pyrene-B (o-phenylenediamine) with a five-membered NBN ring and pyrene-B (1,8-diaminonaphthalene) with a six-membered NBN ring show differing luminescence. Pyrene-B (o-phenylenediamine) shows concentration-dependent luminescence and enhanced emission after grinding at solid state. Pyrene-B (1,8-diaminonaphthalene) exhibits a turn-on type luminescence upon fluoride ion addition at lower concentration, as well as concentration-dependent stability. Further potential applications of Pyrene-B (o-phenylenediamine) on artificial light-harvesting film were demonstrated by using commercial NiR dye as acceptor.

## 1. Introduction

As a dopant, boron has received exceptional attention because of its unique photophysical and redox properties [1,2,3,4,5]. Boron-doped π-systems have received special attentions because of their desirable and potential optical, electronic and sensory properties [6,7,8,9]. Particularly, B-N substitutions in benzene and other cyclic hydrocarbons have been an active and productive field of study for more than 50 years. Many BN-containing organic compounds have been synthesized and applied in pharmacology, electronic materials, and nanoscience [10,11,12,13,14]. Compared with the corresponding all-carbon analogues, organic π-conjugated compounds with a novel BN bond usually have distinct optoelectronic properties/advantages due to the empty p-orbital of the boron center [15,16,17]. Therefore, the replacement of the C=C bond by isoelectronic and isosteric BN units has emerged as a viable strategy to produce functional materials [18,19,20,21,22]. Considerable progress has been made on the comprehensive synthesis of BN- and BNB-doped π-systems with outstanding photophysical properties by pioneering researchers such as Dewar, White, Ashe, Liu, Braunschweig, Schäfer and Jäkle, etc. [23,24,25,26,27,28,29,30,31,32]. However, these outstanding BN- and BNB-doped π-systems suffer from synthesis methods involving rigorous reaction conditions (e.g., air and/or moisture free), multiple steps and unsatisfied total yield [33,34,35,36,37,38].

Recently, NBN units have been incorporated into PAHs, zigzag-edged graphene nanoribbons and organic–inorganic hybrid polymers with the aid of electrophilic aromatic borylation [39,40,41]. In 2019, we reported a simple and efficient synthesis of NBN-doped conjugated diazaborinines by a catalyst-free intermolecular dehydration reaction, and a series of aggregation-induced emissive luminogens have been designed [42]. Nonetheless, the research on the NBN-doped π-conjugated compounds is still very rare. Further investigations on the photophysical and sensory properties of these easily synthesized NBN-doped π-systems will easily expand the functionality and property libraries of BN-doped conjugated compounds, and is therefore highly desirable.

Due to the empty p-orbital of the boron center, boron-doped π-systems also exhibit poor stability, and special molecular designs are required to achieve good stability, such as chemically introducing bulky protection groups. Demonstrating a physically simple method for stabilizing boron-doped π-systems is therefore highly desirable and still very challenging. Meanwhile, when boron-doped π-systems are used for fluoride-ion detection, it is mostly accompanied with turn-off type luminescence disappearance. As is known, there are many factors for achieving luminescence disappearance, which will cause detection interference of fluoride ion with a false report. To realize turn-on type luminescence appearance is an advanced technique in the detection of fluoride ion and is therefore highly desirable [43,44,45,46].

Herein, we demonstrate two pyrene-containing NBN-doped PAHs via a simple synthetic method through efficient intermolecular dehydration between boronic acid and diamine moieties. Their aromaticity, luminescence and sensory properties were investigated. It was found that a physical concentration strategy is a simple method to stabilize boron-doped π-systems.

## 2. Results and Discussion

### 2.1. Fabrication and Aromaticity of NBN-Doped PAHs

**Scheme 1:**
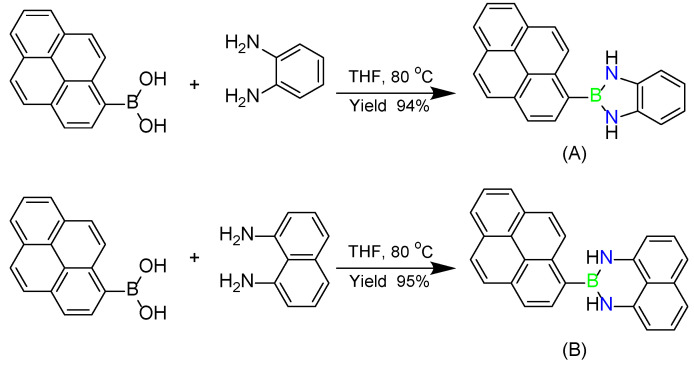
The preparation of NBN-doped pyrene-containing PAHs (**A**) Py-NBN-Ph (**B**) Py-NBN-Naphth through intermolecular dehydration between boronic acid and diamine moieties.

To verify the physical concentration strategy to stabilize boron-doped π-systems, the pyrene moiety was molecularly designed in the NBN-doped PAHs specifically, as shown in Scheme 1. Through one-step intermolecular dehydration between 1-pyrenylboronic acid and o-phenylenediamine or 1,8-diaminonaphthalene in tetrahydrofuran (THF), pyrene-B (1,2-diaminobenzene) (Py-NBN-Ph) with a five-membered NBN ring and pyrene-B (1,8-diaminonaphthalene) (Py-NBN-Naphth) with a six-membered NBN ring were synthesized with a yield of 94% and 95%, respectively. Their successful synthesis was verified by the disappearance of B(OH)_2_ protons at ~8.02 ppm and NH_2_ protons at ~4.37 ppm of 1,2-diaminobenzene and ~5.45 ppm of 1,8-diaminonaphthalene, and by the appearance of NBN protons at ~9.37 ppm of Py-NBN-Ph and ~8.54 ppm of Py-NBN-Naphth in the ^1^H nuclear magnetic resonance (NMR) spectra (Figures S1 and S2). The FI-IR spectra in Figure S4 also confirmed the chemical structure of Py-NBN-Ph and Py-NBN-Naphth. Stable NBN ring formation, strong N- > B dative bonding and hydrogen bonding interactions between B(OH)_2_ and NH_2_ lead to the success of this catalyst-free dehydration with high yield. Meanwhile, as shown in Figure S3, the temperature at 5% weight loss (T_d5%_) of Py-NBN-Ph was 218.3 °C and the T_d5%_ of Py-NBN-Naphth was 345.5 °C, which indicates that the solid power of NBN-doped PAHs has good thermal stability.

**Figure 1:**
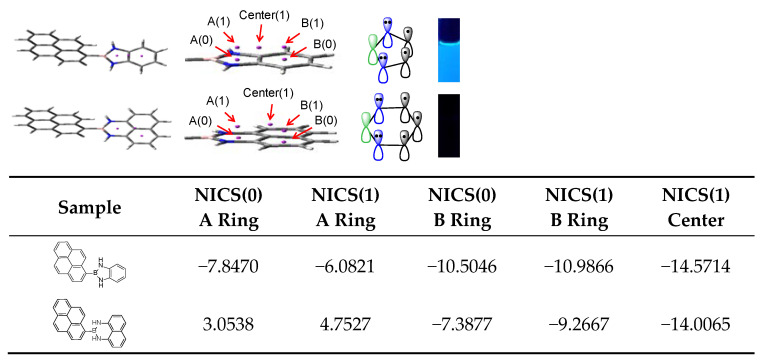
Electron orbital of NBN-doped PAHs, corresponding to the NICS calculation model and NICS values. The photographs in the upper right corner are fluorescent images of Py-NBN-Ph (**up**) and Py-NBN-Naphth (**down**) in THF solution (0.1 mg/mL) under the UV light.

Due to the isoelectronic and isosteric characterization between the B-N and the C=C bond, five- and six-membered NBN rings in Py-NBN-Ph and Py-NBN-Naphth are congeneric to aromatic pyrrole and pyridine, respectively. The aromaticity investigations were therefore carried out via an ab initio method and the nucleus-independent chemical shift (NICS) values were calculated at the GIAO-B3LYP/6-311+G(2d,p) level of the theory at distances of 0.0 Å (A(0) and B(0)) and 1.0 Å (A(1) and B(1)) from each ring system, as well as from the center of the bicyclic systems (Center(1)) in the perpendicular direction, as shown in Figure 1. All rings of Py-NBN-Ph showed negative NICS values, indicating its aromaticity. But Py-NBN-Naphth exhibited positive NICS values for the NBN ring and negative values for other rings and the center, which indicates an anti-aromatic NICS characteristic of NBN ring. The different aromaticity characteristics can be verified by comparing the electron orbits of these two NBN rings. As shown in Figure 1, the number of π-electrons of the five-membered NBN ring in Py-NBN-Ph is 6, indicating an aromaticity characteristic. On the other hand, the number of π-electrons in the six-membered NBN ring of Py-NBN-Naphth is 7 and the extra electron occupies the lowest anti-bonding molecular orbitals, which violates the Hückel rule and results in anti-aromaticity [46,47,48]. Therefore, the five-membered NBN ring in Py-NBN-Ph and six-membered NBN ring in Py-NBN-Naphth exhibit totally different aromaticity. The electronic distribution of HOMO and LUMO orbitals of Py-NBN-Ph and Py-NBN-Naphth was calculated and shown in Figure S9 and Table S3.

**Figure 2:**
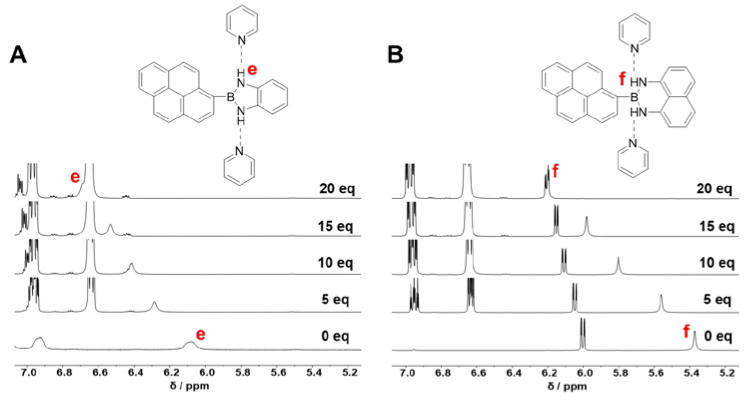
^1^H-NMR titration of (**A**) Py-NBN-Ph (c_0_ = 1.572 × 10^−3^ M) and (**B**) Py-NBN-Naphth (c_0_ = 8.152 × 10^−3^ M) with pyridine in C_6_D_6_ at 298 K.

The NH proton in BN and NBN isostere is more acidic than the CH analogues, and could engage in to H-bonding interactions. Thus, Py-NBN-Ph and Py-NBN-Naphth could act as H-bonding donors. We investigated the H-bonding complex between them and pyridine by ^1^H NMR. Upon addition of increasing amount of pyridine (up to 20 equiv) to a solution of Py-NBN-Ph in C_6_D_6_, a downfield shift from 6.08 ppm to 6.62 ppm of the H (e) proton was observed, as shown in Figure S10A and Figure 2A. This indicates that H-bonding formation occurs between the H atom of -NH in NBN ring and the N atom of the pyridine. The same phenomenon was obtained by titrating Py-NBN-Naphth (c_0_ = 8.152 × 10^−3^ M) with pyridine (up to 20 equiv) in C_6_D_6_, and a downfield shift from 5.38 ppm to 6.21 ppm of the H (f) proton was observed, as shown in Figure S10B and Figure 2B. H-bonded complexes of pyridine·Py-NBN-Ph, pyridine·Py-NBN-Naphth were formed, proving the good H-bonding capabilities of the NBN functional group. These findings provide a new idea for the application of NBN-doped PAHs in supramolecular chemistry.

### 2.2. Luminescent Properties of NBN-Doped PAHs

**Figure 3:**
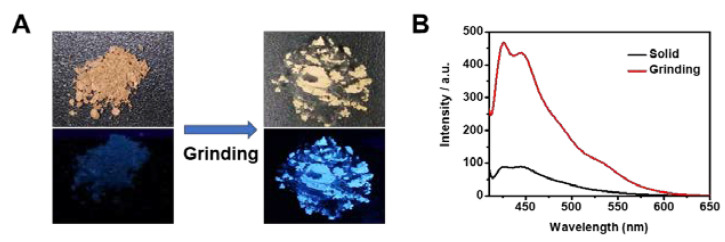
(**A**) Digital images of Py-NBN-Ph at a solid state and after grinding (under sunlight and UV light at 365 nm); (**B**) Emission spectra at a solid state before and after grinding (excited at 398 nm).

Boron-doped π-systems are widely utilized as luminogens and sensors accompanied with luminescence changes attributed to the empty p-orbital of the boron center in π-systems. As is known, pyrene is a well-investigated luminescent material [49,50,51]. Therefore, the luminescent behaviour of pyrene-containing NBN-doped PAHs was investigated. The absorption spectra of Py-NBN-Ph and Py-NBN-Naphth were similar and shown in Figure S5. Interestingly, Py-NBN-Ph and Py-NBN-Naphth exhibited totally different luminescent properties in solution, as shown in Figure 1. Py-NBN-Ph showed a strong cyan fluorescence, with a maximum emission wavelength of 460 nm in solution. By contrast, Py-NBN-Naphth was nonemissive both in solution (such as in THF, methanol) and at a solid state, which may have been caused by the antiaromaticity-type NICS values and the extra electron on the lowest anti-bonding molecular orbitals of the NBN ring. These results indicate that pyrene-containing NBN-doped PAHs exhibits aromaticity-dependent luminescence, and the difference in luminescence derives from the significant differences in aromaticity mentioned above. Meanwhile, Py-NBN-Ph is weakly emissive at a solid state due to the aggregation-caused quenching (ACQ) effect, as shown in Figure S6 [52,53]. Interestingly, it exhibited grinding enhanced emission behavior. As shown in Figure 3, Py-NBN-Ph showed strong blue emission after grinding, exhibiting amorphization-induced emission behavior, which conforms to reports in the literature on pyrene-containing material [54].

### 2.3. Fluoride Ion Detection

**Figure 4:**
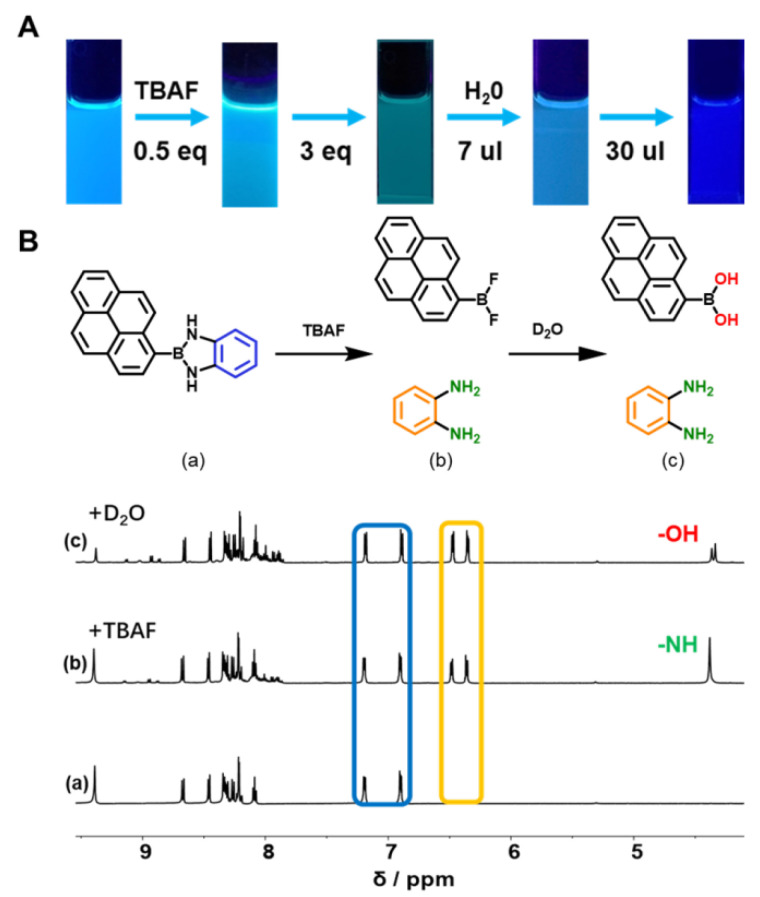
(**A**) Emission changes of Py-NBN-Ph solution after adding TBAF and water; (**B**) ^1^H NMR spectra of Py-NBN-Ph in DMSO-*d_6_* (**a**), after the addition of 1.0 equal of TBAF (**b**), and followed by the addition of 15 μL of D_2_O (**c**).

**Figure 5:**
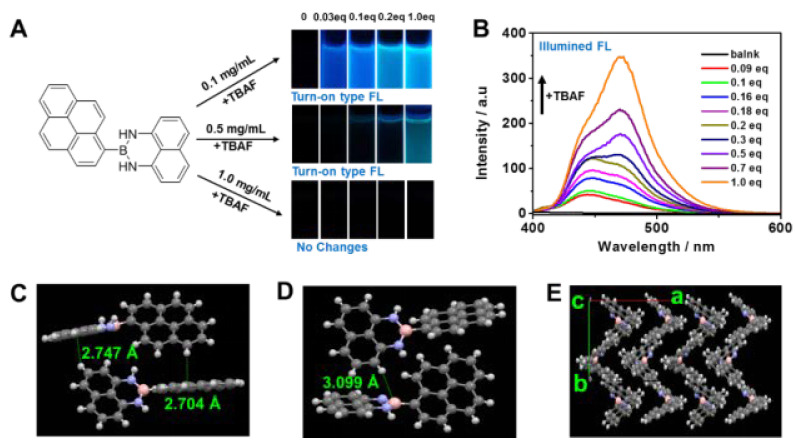
The responsive behaviors of Py-NBN-Naphth. (**A**) Schematic diagram with the addition of fluoride ion at different concentrations; (**B**) Emission spectra with the addition of different amounts of TBAF; (**C**–**E**) Single-crystal structures.

Another interesting aspect is the aromaticity and concentration-dependent sensory properties of these pyrene-containing NBN-doped PAHs. The utilization of these two materials for fluoride ion detection was carried out, and different phenomena were exhibited (Figure 4, Figure 5 and Figure S7). For Py-NBN-Ph, it was highly emissive at 460 nm with a cyan color in THF (1 mg/mL). With the addition of tetrabutylammonium fluoride (TBAF) to three equivalents, the luminescent intensity decreased by 85%. The emission was almost quenched, and fell to less than 5% intensity when 6 equivalent fluoride ions were added. Further addition of water caused the recovery of cyan emission and then a shift to a blue color, as shown in Figure 4A. The whole process was verified by ^1^H NMR titrations in Figure 4B, where NBN unit changed to pyrene-BF_2_ with the addition of fluoride ion and then to pyrene-B(OH)_2_ with the addition of water. Py-NBN-Ph exhibited a turn-off type luminescence in the fluoride ion detection.

In comparison, for Py-NBN-Naphth, 1 mg/mL solution in THF showed no changes with the addition of fluoride ion or water, which can be verified from photo images in Figure 5A. The 1 mg/mL solution of Py-NBN-Naphth in THF was stable with fluoride ion, and its ^1^H and ^11^B NMR spectra before and after the addition of fluoride ion exhibited no changes, as shown in Figure S8. In addition, the Py-NBN-Naphth solution was stable at 1 mg/mL, even with the addition of concentrated HCl (11.8 mol/L) solution, which was verified by ^1^H spectra without any changes. However, when the concentration of Py-NBN-Naphth decreased to 0.1 mg/mL, it exhibited a turn-on type luminescence upon the addition of fluoride ion. Although the 0.1 mg/mL solution of Py-NBN-Naphth was non-emissive, the emission appeared and its strength increased with the addition of fluoride ion, as shown in Figure 5A,B. The 0.5 mg/mL solution of Py-NBN-Naphth also showed a turn-on type luminescence when 1.0 equivalent fluoride ion was added. All these results indicate that stability and sensory properties of Py-NBN-Naphth depend on its concentration. To investigate the mechanism for these concentration-dependent properties, its single crystal structures were revealed. As shown in Figure 5C–E, the boron center is embedded by pyrene and naphthalene cycles, and there are weak intermolecular B···H interactions with a distance of 3.1 Å as well. Based on the existence of an embedding effect and intermolecular B···H interactions, we assume that they protect the boron center from fluoride ion and acid when the concentration is high, while these embedding effects and weak interactions can be ignored at lower concentration, resulting in the concentration-dependent stability and sensory properties.

### 2.4. Artificial Light-Harvesting Film

**Figure 6:**
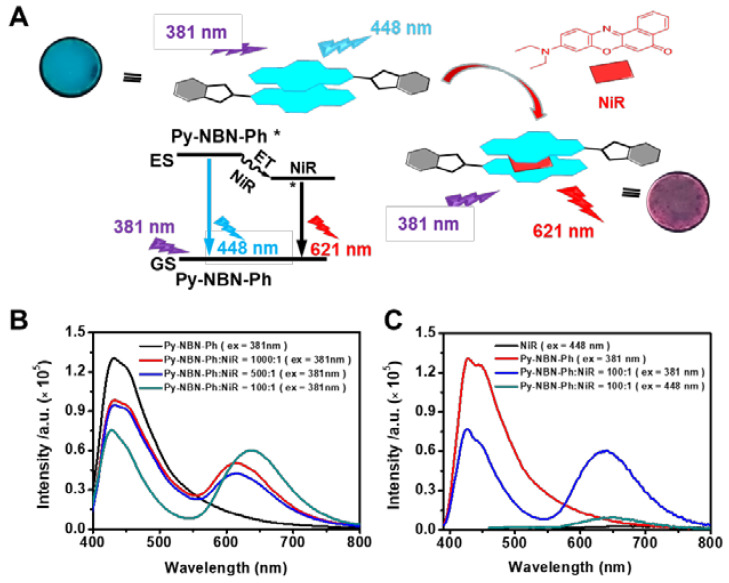
Artificial light-harvesting application of Py-NBN-Ph. (**A**) Schematic diagram of light-harvesting application, Py-NBN-Ph is the ground state molecule and Py-NBN-Ph* is the excited state molecule; (**B**) Emission spectra of light-harvesting films with different Py-NBN-Ph/NiR ratios; (**C**) Emission spectra of NiR, Py-NBN-Ph and Py-NBN-Ph/NiR (100:1).

To demonstrate the applications of pyrene-containing NBN-doped PAHs, the artificial light-harvesting film was prepared with Py-NBN-Ph as donor and nile red (NiR) as acceptor, because the absorption of the NiR acceptor overlaps with the emission of the Py-NBN-Ph donor [55,56,57,58]. The artificial light-harvesting films were prepared with different Py-NBN-Ph/NiR ratios. As shown in Figure 6B, the luminescent intensity at 621 nm assigned to the NiR acceptor increased with the increase of the NiR ratio, and the luminescent intensity at 448 nm assigned to the Py-NBN-Ph donor decreased under the excitation at 381 nm, indicating successful energy transfer from Py-NBN-Ph at an excited state (Py-NBN-Ph*) to NiR at a ground state. The energy transfer can be further verified by comparing the luminescent intensities of different control experiments (Figure 6C). In the absence of Py-NBN-Ph, the NiR acceptor emits weakly, with low luminescent intensity (black line in Figure 6C). The Py-NBN-Ph donor without NiR emits at 448 nm under the excitation at 381 nm, as the red line in Figure 6C shows. In the presence of Py-NBN-Ph (Py-NBN-Ph/NiR ratio of 100/1), the energy transfer between the Py-NBN-Ph donor and the NiR acceptor occurs with the decrease in the luminescent intensity of Py-NBN-Ph and the increase in the luminescent intensity of NiR.

The photophysical process of this artificial light-harvesting film is therefore illustrated in Figure 6A. When the artificial light-harvesting film absorbs the photo energy at 381 nm, the Py-NBN-Ph donor at the ground state will be excited to the excited state as Py-NBN-Ph*, which is supposed to return to the ground state radiatively with emission at 448 nm. In the presence of NiR, the Py-NBN-Ph* transfers energy to the NiR molecules at the ground state, resulting in the formation of NiR* at an excited state. Finally, the NiR* molecules radiatively return to the ground state with an emission at 621 nm.

## 3. Materials and Methods

### 3.1. Materials

1-pyrenylboronic acid, 1,2-phenylenediamine, 1,8-diaminonaphthalene, THF and TBAF were purchased from Aladdin (China) and used without further purification. THF was distilled from sodium/benzophenone prior to use. Ultra-pure water treated by a water purification system was used. All other reagents and solvents were purchased as analytical grade from Energy Chemical (China) and used without further purification.

### 3.2. Synthetic Procedures

#### 3.2.1. Synthesis of Py-NBN-Ph

1-pyrenylboronic acid (0.246 g, 1 mmol) and 1,2-phenylenediamine (0.108 g, 1 mmol) were dissolved in THF (10 mL), and the reaction solution was heated from room temperature to 80 °C to reflux and continued for 12 h under nitrogen protection. The reaction was stopped and cooled, then the solution was concentrated by a rotary evaporator under reduced pressure and the crude product was purified by column chromatography on silica gel (petroleum ether: ethyl acetate = 10:1) to yield Py-NBN-Ph. ^1^H NMR (600 MHz, DMSO-*d_6_*) δ (ppm) 6.89 (dd, J = 4.8, 4.92 Hz, 2H, -C_6_H_4_), 7.20 (dd, J = 4.86, 4.92 Hz, 2H, -C_6_H_4_), 9.37 (s, 2H, -NH). ^11^B NMR (600MHz, DMSO-*d_6_*) δ (ppm) 28.21.

#### 3.2.2. Synthesis of Py-NBN-Naphth

Py-NBN-Naphth was prepared by a similar procedure as the above, except using 1,8-diaminonaphthalene (0.158 g, 1 mmol) instead of 1,2-phenylenediamine. ^1^H NMR (600 MHz, DMSO-*d_6_*) δ (ppm) 6.62(d, J = 10.98 Hz, 1H, -C_6_H_4_), 7.03 (d, J = 12.3 Hz, 1H, -C_6_H_4_), 7.17 (t, J = 23.34 Hz, 2H, -C_6_H_4_), 8.54 (s, 2H, -NH). ^11^B NMR (600MHz, DMSO-*d_6_*) δ (ppm) 30.22.

#### 3.2.3. Ab Initio Calculations

All the molecules were fully optimized by density functional theory (DFT) methods with the B3LYP hybrid functional and the 6-311+G(d,p) basis set implemented in the Gaussian 09 package (Gaussian, Inc., Wallingford, CT, USA). The values of nucleus-independent chemical shift (NICS) were simulated with the gauge-independent atomic orbital (GIAO) method. The environment layer was treated with the universal force field (UFF). All the atoms were allowed to relax on the ground.

#### 3.2.4. The Detection of TBAF

The THF solutions of Py-NBN-Ph and Py-NBN-Naphth with concentrations of 1 mg/mL, 0.5 mg/mL and 0.1 mg/mL were used to detect TBAF. The fluorescence spectra upon different amounts of TBAF addition were recorded.

#### 3.2.5. Thin-Film Fabrication

The Py-NBN-Ph was dissolved in THF at a concentration of 10 mg/mL. Ten microliters of this solution was deposited on a quartz glass sheet. Then it was put in a Petri dish and left undisturbed until the solvent evaporated at room temperature.

### 3.3. Characterizations

NMR spectroscopy. The NMR measurements were performed on a Bruker Ascend TM ECZ600S spectrometer (Bruker, Rheinstetten, Germany) in DMSO-*d_6_* using tetramethylsilane as an internal standard.

UV-vis absorption. The spectra were recorded in THF on a Shimadzu UV-2450 UV-Vis spectrophotometer (Shimadzu, Kyoto, Japan) at room temperature.

Fluorescence spectroscopy. The spectra were performed on a RF-5301pc fluorescence spectrophotometer (Shimadzu, Kyoto, Japan) in a quartz cuvette with a path length of 1 cm.

Fourier transform infrared (FT-IR) spectroscopy. FT-IR data were obtained from Bruker VERTEX 70 (Bruker, Rheinstetten, Germany). The spectra were recorded from an accumulation of 32 scans in the range of 4000~400 cm^−^^1^.

## 4. Conclusions

In summary, we demonstrated unpredicted concentration-dependent sensory properties of pyrene-containing NBN-doped PAHs via a simple synthetic method through efficient intermolecular dehydration between boronic acid and diamine moieties. Py-NBN-Ph with a five-membered NBN ring exhibits an aromaticity-type NICS characteristic with traditional ACQ luminescent properties and grinding enhanced emission at a solid state, while Py-NBN-Naphth with a six-membered NBN ring exhibits an antiaromaticity-type NICS characteristic without luminescence. Py-NBN-Naphth exhibits unpredicted concentration-dependent stability and turn-on type luminescent sensory properties towards fluoride ion. A further potential application of NBN-doped pyrene-containing PAH was demonstrated as well by using Py-NBN-Ph as donor and commercial NiR dye as acceptor. This work therefore opens up a new avenue for realizing the luminescent and sensory properties of boron-doped PAHs through a simple synthetic method, and also expands the property and application libraries of BN-doped π-systems.

## Data Availability

The data presented in this study are available on request from the corresponding authors.

## Supplementary Materials

The following supporting information can be downloaded online, Figure S1: ^1^H (A), ^13^C (B) and ^11^B (C) NMR spectra of Py-NBN-Ph in DMSO-d_6_; Figure S2: ^1^H (A), ^13^C (B) and ^11^B (C) NMR spectra of Py-NBN-Naphth in DMSO-d_6_; Figure S3: The TGA curves of Py-NBN-Ph (A) and Py-NBN-Naphth (B); Figure S4: FT-IR spectra of Py-NBN-Ph (A) and Py-NBN-Naphth (B); Figure S5: The absorption spectra of Py-NBN-Ph (A) and Py-NBN-Naphth (B); Figure S6: Fluorescence properties of Py-NBN-Ph. (A) Emission spectra in THF mixtures with different concentration (excited at 384 nm); (B) Plot of relative emission intensity at 460 nm and the corresponding fluorescence diagram; (C) Photographic images of one drop of Py-NBN-Ph solution on thin-layer chromatography under UV lamp @ 365 nm; (D) Digital photos of Py-NBN-Ph (0.4 mg/mL) in H_2_O/THF mixtures with different H_2_O fractions (vol %) under UV lamp @ 365 nm (up) and under daylight (down); Figure S7: Fluoride ion detection of Py-NBN-Ph (1 mg/mL in THF). (A) Emission spectra with different amount of TBAF (excitation at 356 nm); (B) Plot of relative emission intensity at 460 nm with amounts of TBAF (excitation at 356 nm); (C) Emission spectra with further addition of water (excitation at 356 nm); Figure S8: The NMR spectra of Py-NBN-Naphth. (A) ^1^H NMR spectra and (B) ^11^B NMR spectra with and without TBAF; (C) ^1^H NMR spectra with and without DCl; Figure S9: The electronic distribution of HOMO and LUMO orbitals of Py-NBN-Ph and Py-NBN-Naphth; the electronic distribution of HOMO orbitals of Py-NBN-Ph (A), the electronic distribution of LUMO orbitals of Py-NBN-Ph (B), the electronic distribution of HOMO orbitals of Py-NBN-Naphth (C), the electronic distribution of LUMO orbitals of Py-NBN-Naphth (D); Figure S10: ^1^H NMR spectra of Py-NBN-Ph (A) and Py-NBN-Naphth (B) in C_6_D_6_. Table S1: The elemental analysis of Py-NBN-Ph and Py-NBN-Naphth; Table S2: Crystal data and structure refinement for NBN-doped pyrene-containing PAH with six-membered NBN ring; Table S3: The HOMO energy and LUMO energy of Py-NBN-Ph and Py-NBN-Naphth.

## Sample Availability

Samples of the compounds are available from the authors.
