# Supplementary material for: Unpredicted Concentration-Dependent Sensory Properties of Pyrene-Containing NBN-Doped Polycyclic Aromatic Hydrocarbons
Source: Molecules. 2022 Jan 5;27(1):327. doi: 10.3390/molecules27010327 (PMC8746585; doi:10.3390/molecules27010327)
Supplement: Supplementary file 1 [file molecules-27-00327-s001.zip › molecules-1491217-supplementary.pdf]

Supporting Information

Unpredicted Concentration-Dependent Sensory Properties of Pyrene-Containing NBN-Doped Polycyclic Aromatic Hydrocarbons

Hang Xiao [1,2,†], Tao Li [1,2,†], Xiao-Li Sun [1,*], Wen-Ming Wan [2,3,*], Hongli Bao [2], Qingrong Qian [1] and Qinghua Chen [1]

1 Fujian Key Laboratory of Pollution Control &Resource Reuse, , Engineering Research Center of Polymer Green Recycling of Ministry of Education, College of Environmental Science and Engineering, Fujian Normal University, Fuzhou 350007, China; QBX20210117@yjs.fjnu.edu.cn (H.X.); qsz20191180@student.fjnu.edu.cn (T.L.); qrqian@fjnu.edu.cn (Q.Q.); cqhuar@126.com (Q.C.)

2 State Key Laboratory of Structural Chemistry, Key Laboratory of Coal to Ethylene Glycol and Its ReLated Technology, Center for Excellence in Molecular Synthesis, Fujian Institute of Research on the Structure of Matter, Chinese Academy of Sciences, 155 West Yangqiao Road, Fuzhou 350002, China; hlbao@fjirsm.ac.cn

3 State Key Laboratory of Molecular Engineering of Polymers, Fudan University, Shanghai 200438, China

* Correspondence: sunxiaoli@fjnu.edu.cn (X.S.); wanwenming@fjirsm.ac.cn (W.W.)

† These authors contributed equally to this work.

Supporting information for this article is given via a link at the end of the document.

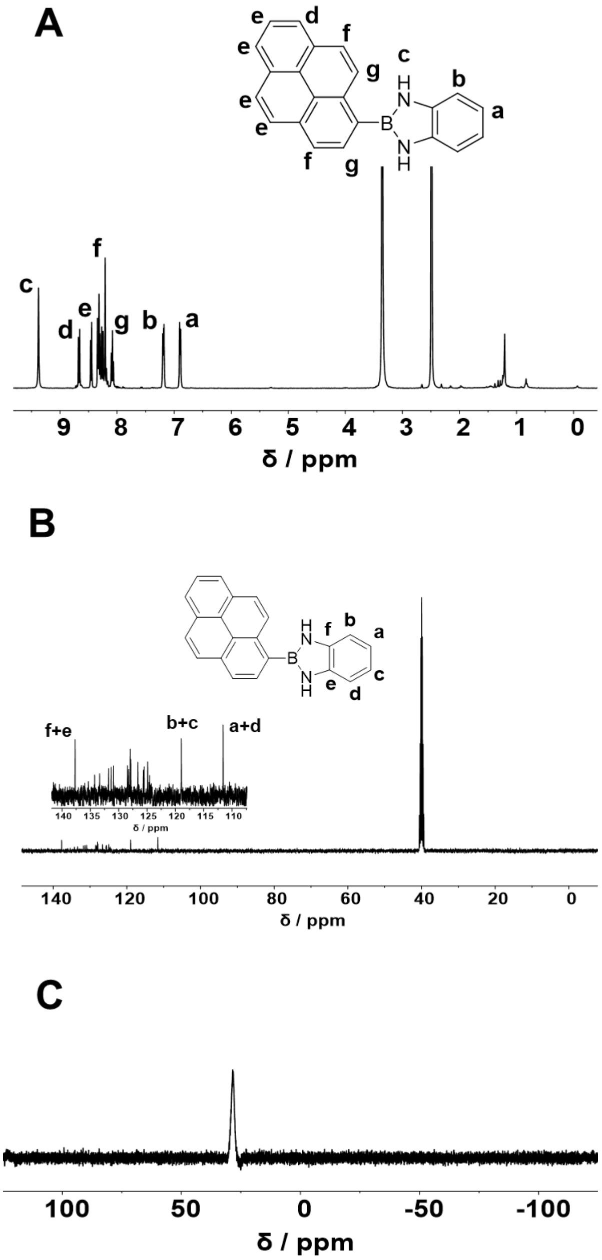

Figure S1. ^{1}H (A), ^{13}C (B) and ^{11}B (C) NMR spectra of Py-NBN-Ph in DMSO-d_6.

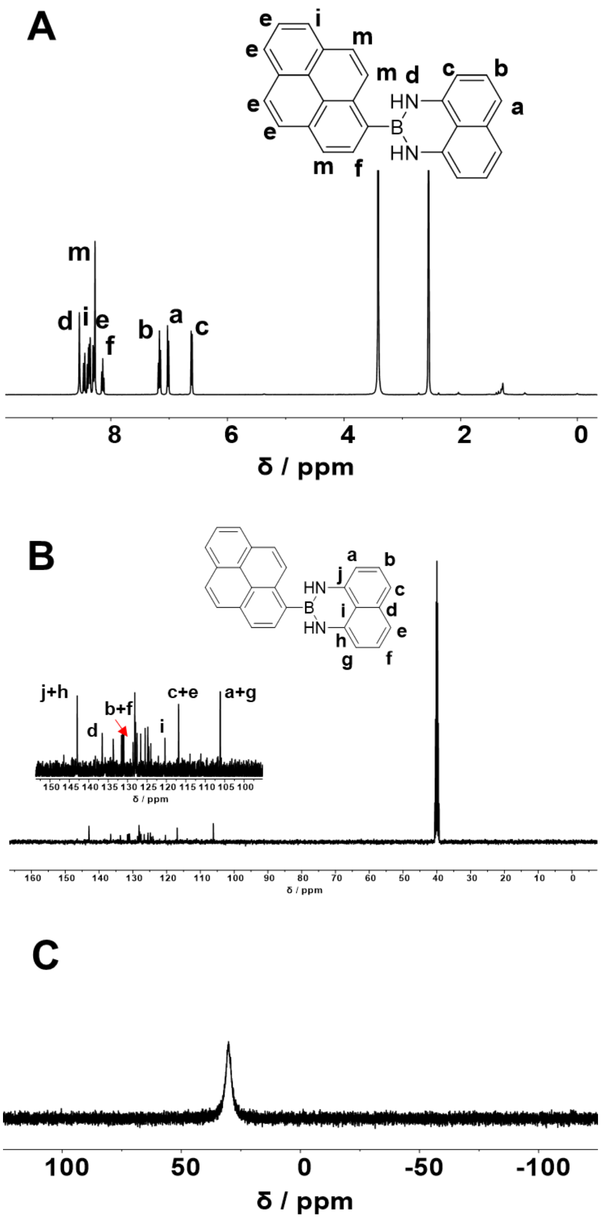

Figure S2. ^{1}H (A), ^{13}C (B) and ^{11}B (C) NMR spectra of Py-NBN-Naphth in DMSO-d_6.

Table S1. The elemental analysis of Py-NBN-Ph and Py-NBN-Naphth

Sample	N Area	C Area	H Area	N(%)	C(%)	H(%)	C/N	C/H
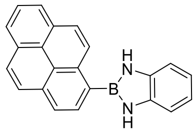	**5.340**	**39.653**	**7.680**	**7.90**	**82.54**	**4.851**	**10.4505**	**17.01**
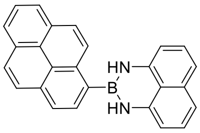	**5.019**	**39.996**	**7.025**	**7.41**	**84.57**	**4.510**	**11.4139**	**18.75**

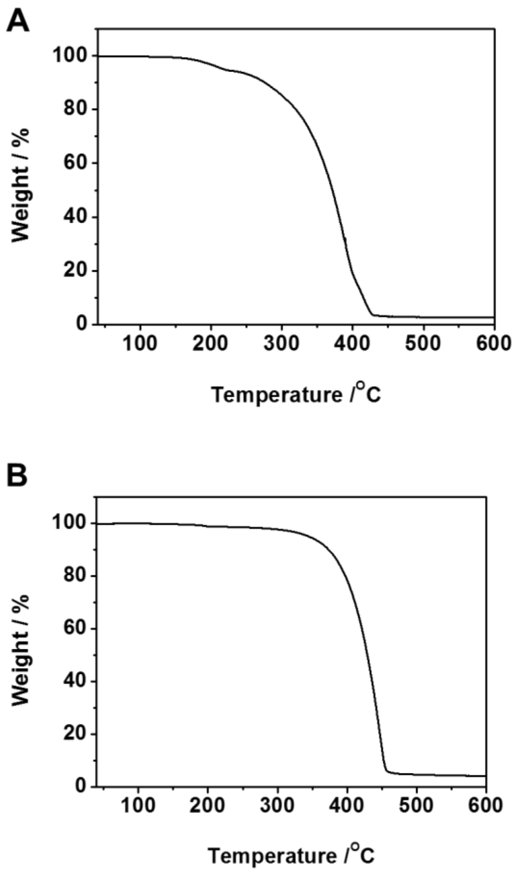

Figure S3. The TGA curve of Py-NBN-Ph (A. The temperature at 5% weight loss (T_{d5}) of was 218.3 °C) and Py-NBN-Naphth (B. The temperature at 5% weight loss (T_{d5}) was 345.5 °C)

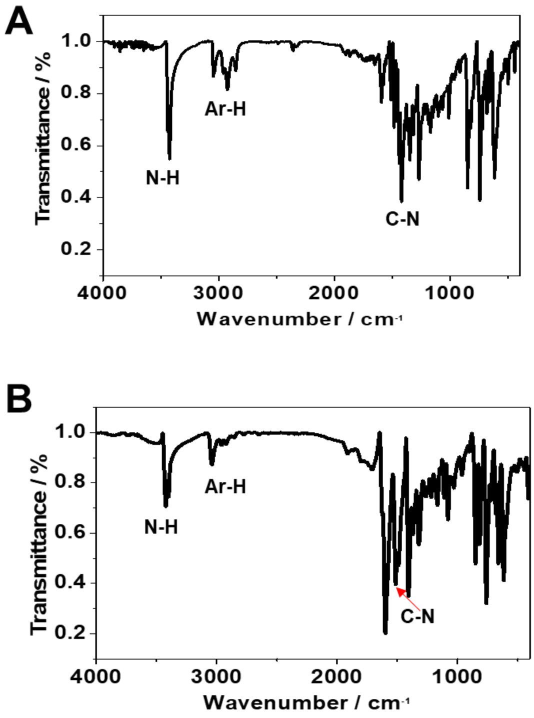

Figure S4. FT-IR spectra of Py-NBN-Ph (A) and Py-NBN-Naphth (B).

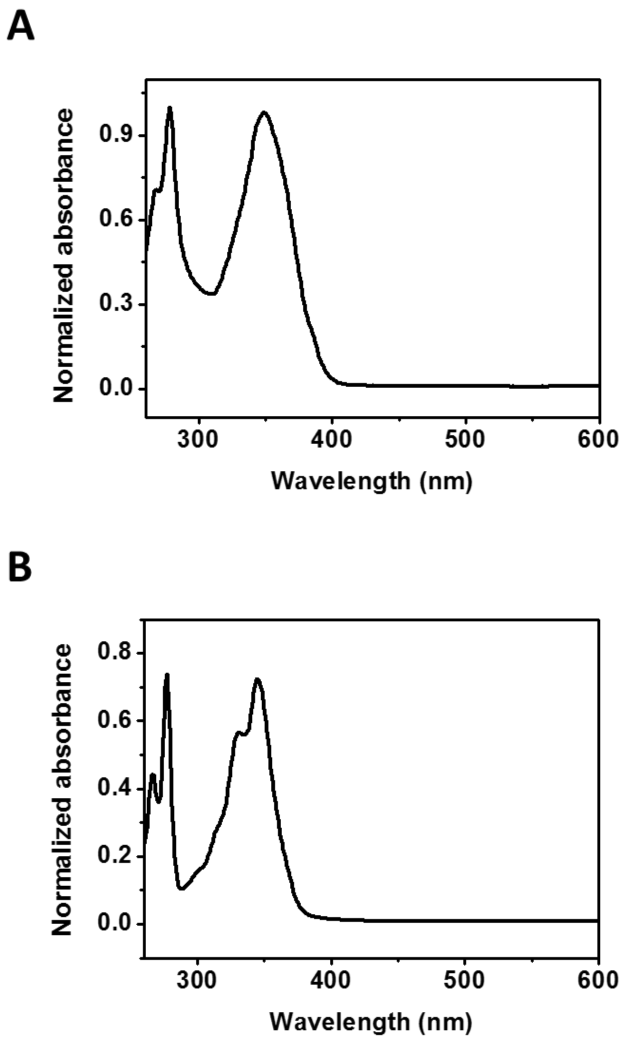

Figure S5. The absorption spectra of Py-NBN-Ph (A) and Py-NBN-Naphth (B).

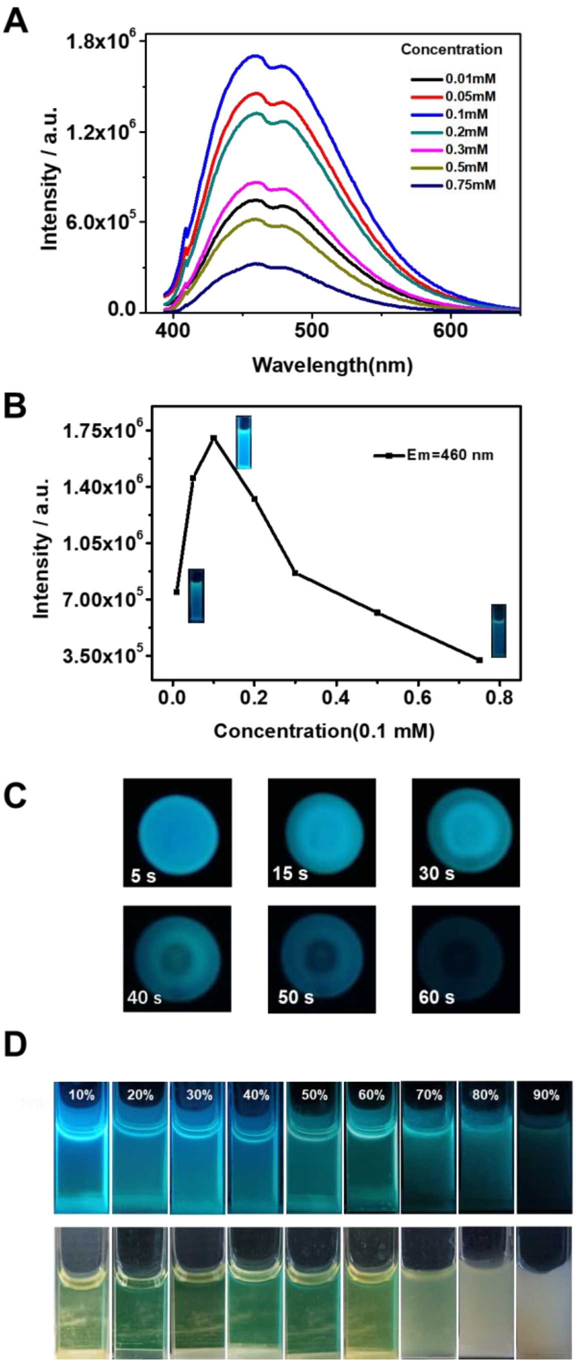

Figure S6. Fluorescence properties of Py-NBN-Ph. (A) Emission spectra in THF mixtures with different concentration (excited at 384 nm); (B) Plot of relative emission intensity at 460 nm and the corresponding fluorescence diagram; (C) Photographic images of one drop of Py-NBN-Ph solution on thin-layer chromatography under UV lamp @ 365 nm; (D) Digital photos of Py-NBN-Ph (0.4 mg/mL) in H_2O/THF mixtures with different H_2O fractions (vol %) under UV lamp @ 365 nm (up) and under daylight (down).

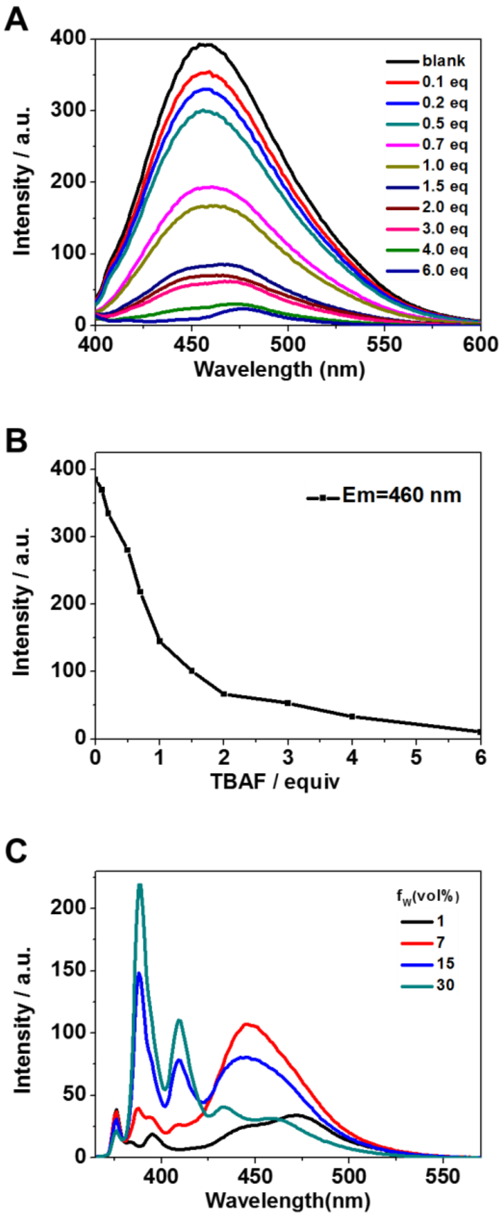

Figure S7. Fluoride ion detection of Py-NBN-Ph (1 mg/mL in THF). (A) Emission spectra with different amount of TBAF (excitation at 356 nm); (B) Plot of relative emission intensity at 460 nm with amounts of TBAF (excitation at 356 nm); (C) Emission spectra with further addition of water (excitation at 356 nm).

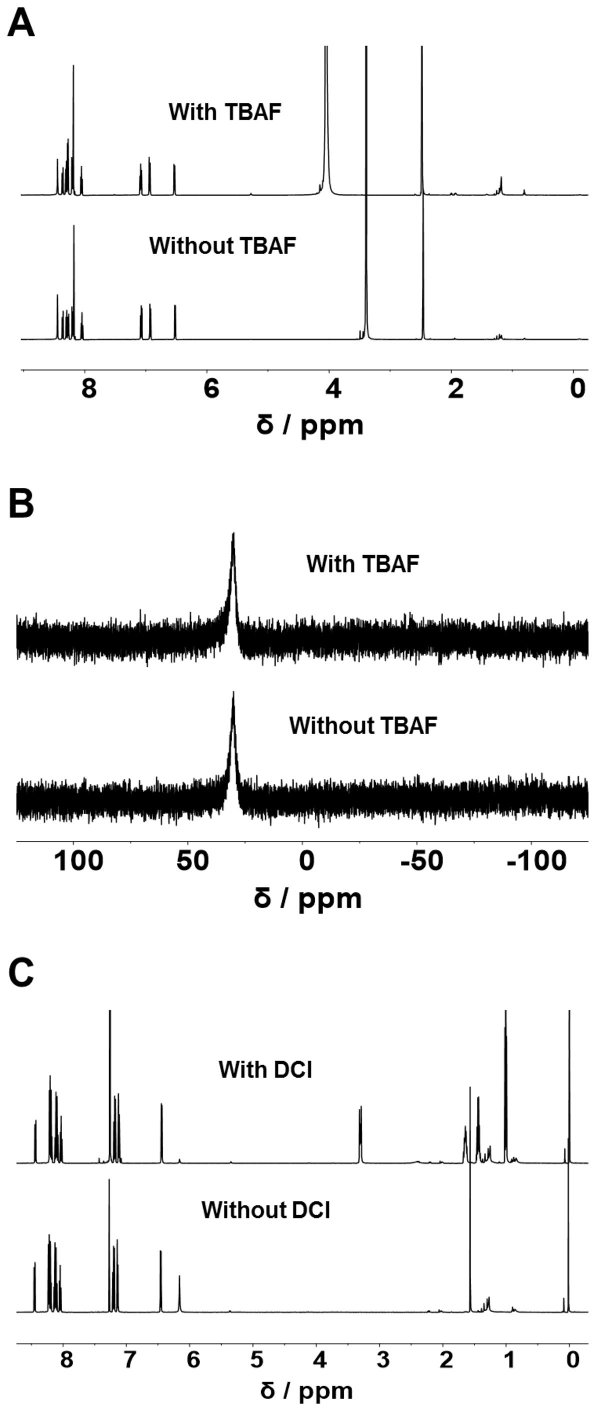

Figure S8. The NMR spectra of Py-NBN-Naphth. (A) ^{1}H NMR spectra and (B) ^{11}B NMR spectra with and without TBAF; (C) ^{1}H NMR spectra with and without DCl.

Single crystal data

Table S2. Crystal data and structure refinement for NBN-doped pyrene-containing PAH with six-membered NBN ring.

CCDC	1986343	
Identification code	lt-21	
Empirical formula	$C_{26}H_{17}BN_2$	
Formula weight	368.22	
Temperature	99.9(4) K	
Wavelength	1.3405 Å	
Crystal system	Monoclinic	
Space group	P 1 21 1	
Unit cell dimensions	a = 14.8631(4) Å	α= 90°.
	b = 8.50350(10) Å	β= 107.360(3)°.
	c = 14.9338(4) Å	γ= 90°.
Volume	1801.48(8) $Å^3$	
Z	4	
Density (calculated)	1.358 Mg/m^3	
Absorption coefficient	0.386 mm^{-1}	
F(000)	768	
Crystal size	0.5 x 0.4 x 0.01 mm^3	
Theta range for data collection	2.695 to 60.708°.	
Index ranges	-19<=h<=19, -9<=k<=11, -19<=l<=19	
Reflections collected	20824	
Independent reflections	6825 [R(int) = 0.1047]	
Completeness to theta = 53.543°	99.9 %	
Absorption correction	Semi-empirical from equivalents	
Max. and min. transmission	1.00000 and 0.68495	
Refinement method	Full-matrix least-squares on F^2	
Data / restraints / parameters	6825 / 1 / 524	
Goodness-of-fit on F^2	1.088	
Final R indices [I>2sigma(I)]	R1 = 0.0726, wR2 = 0.1675	
R indices (all data)	R1 = 0.0845, wR2 = 0.1743	
Absolute structure parameter	0.7(12)	
Extinction coefficient	n/a	
Largest diff. peak and hole	0.586 and -0.575 e. $Å^{-3}$	

Table S3. The HOMO energy and LUMO energy of Py-NBN-Ph and Py-NBN-Naphth

Sample	HOMO (eV)	LUMO (eV)	Eg (eV)
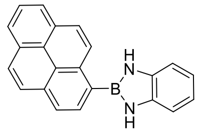	**-5.41008**	**-1.85178**	**3.55830**
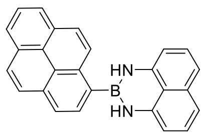	**-4.96509**	**-1.90917**	**3.05592**

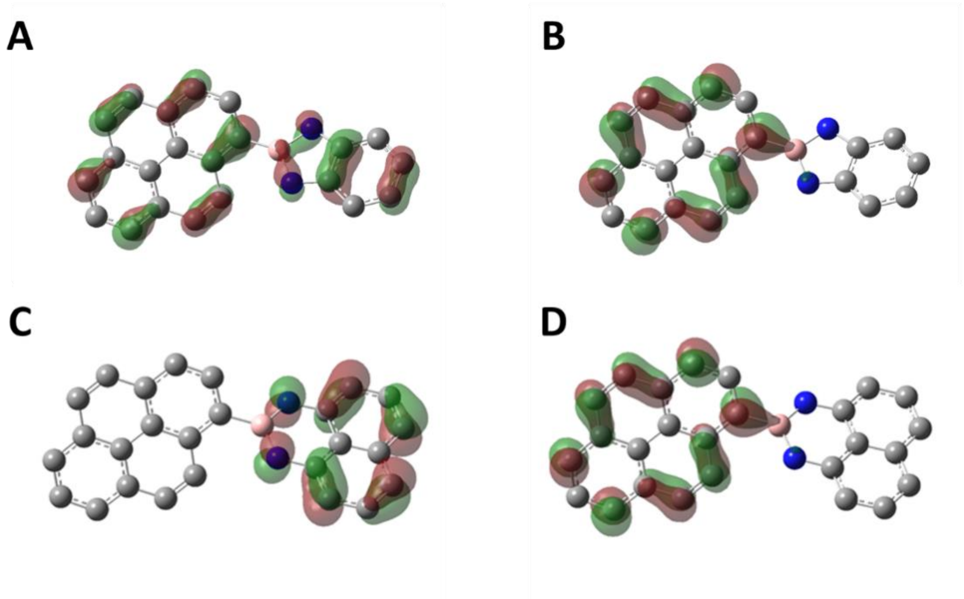

Figure S9. The electronic distribution of HOMO and LUMO orbitals of Py-NBN-Ph and Py-NBN-Naphth; the electronic distribution of HOMO orbitals of Py-NBN-Ph (A), the electronic distribution of LUMO orbitals of Py-NBN-Ph (B), the electronic distribution of HOMO orbitals of Py-NBN-Naphth (C), the electronic distribution of LUMO orbitals of Py-NBN-Naphth (D).

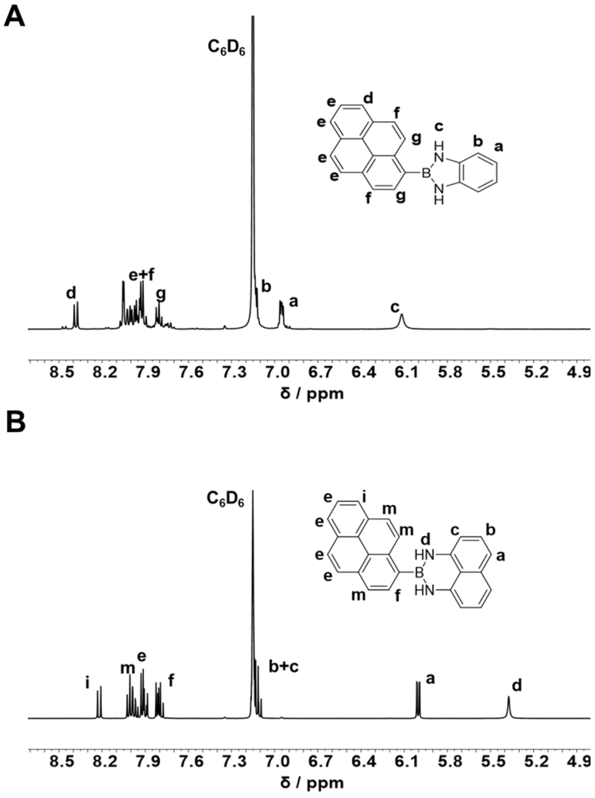

Figure S10. ^{1}H NMR spectra of Py-NBN-Ph (A) and Py-NBN-Naphth (B) in C_6D_6.
